# Subcapsular Splenic Hematoma After Diagnostic Colonoscopy: A Case Report

**DOI:** 10.7759/cureus.69850

**Published:** 2024-09-21

**Authors:** Shawn Howell, Taylor Barton, Idean A Pourshams, Christopher Eckman

**Affiliations:** 1 Internal Medicine, University of Arkansas for Medical Sciences, Fayetteville, USA; 2 Internal Medicine, Mercy Hospital, Rogers, USA

**Keywords:** acute blood loss anemia, colonoscopy, colonoscopy complication, splenic hematoma, splenic injury

## Abstract

Colonoscopy is a widely performed diagnostic and therapeutic procedure essential for the screening, diagnosis, and management of various colorectal conditions. It is a routine and relatively safe procedure. Unfortunately, sometimes complications arise, one of the rarest being splenic injury. A splenic hematoma following colonoscopy can lead to significant morbidity and can be fatal if not promptly recognized and managed. We report a case of a 58-year-old female who initially refused imaging after presenting to the ED with abdominal pain following her procedure, but then presented again days later and was found to have a splenic hematoma and hemoperitoneum. She was ultimately managed medically and after an uneventful hospital course and was able to be discharged without requiring a splenectomy.

## Introduction

Colonoscopy is a widely performed diagnostic and therapeutic procedure essential for the screening, diagnosis, and management of various colorectal conditions. Despite its relative safety, colonoscopy is associated with several potential complications, such as bleeding (0.59%) [1], perforation (0.07%) [1], and, rarely, splenic injury. The first case of splenic injury following colonoscopy was described in a report in 1974 [2]. Since then, multiple case reports have been published demonstrating this complication [2-16]. 

Splenic injury is thought to be caused by either direct trauma from the colonoscope as it traverses the splenic flexure or traction on the splenocolic ligament during the procedure. Risk factors for splenic injury include older age, female sex, splenomegaly, adhesions from prior surgery, malignancy, polypectomy, and ongoing anticoagulation [3]. Following injury, patients typically experience symptoms hours after the procedure and most ultimately require a splenectomy. Some splenic injuries are mild and require observation without surgical intervention, such as in this case. Splenic hematoma following colonoscopy is an exceedingly uncommon complication, occurring in approximately one out of every 5882 colonoscopies, and with a mortality rate of 5%. If not promptly recognized and managed, it can lead to significant morbidity [8].

## Case presentation

A 58-year-old female with inactive, uncomplicated Crohn's disease, treated with adalimumab, hypothyroidism, and a history of cholecystectomy, Cesarean section, and hysterectomy underwent an esophagogastroduodenoscopy and colonoscopy for symptoms of epigastric abdominal pain, anorexia, nausea, and vomiting. The colonoscopy was uncomplicated and straightforward. Endoscopic findings from the procedures were normal and biopsies taken revealed mildly active chronic gastritis, preserved villous architecture of the duodenum, normal ileal mucosa, and normal colonic mucosa. On the same day of the procedures, she presented to the emergency department (ED) with acute worsening of her abdominal pain. She was afebrile and hemodynamically stable. Physical exam revealed mild generalized abdominal tenderness. Labs were notable for a normal Hemoglobin of 13.5, unremarkable complete metabolic panel, normal lipase, and an elevated C-reactive protein of 20. The patient was offered computed tomography (CT) imaging of the abdomen and pelvis but declined and was discharged home with a prescription for hyoscyamine and ondansetron. She was instructed to return to the ED if her symptoms continued to worsen. She again presented two days later with similar complaints along with shortness of breath and a nonproductive cough. She was febrile with a temperature of 103.2°F, tachycardic up to 124 beats per minute(bpm), and hypotensive with a blood pressure of 85/50 and a mean arterial pressure of 62. Her physical exam revealed generalized tenderness to palpation without rebound or guarding.

**Figure 1:**
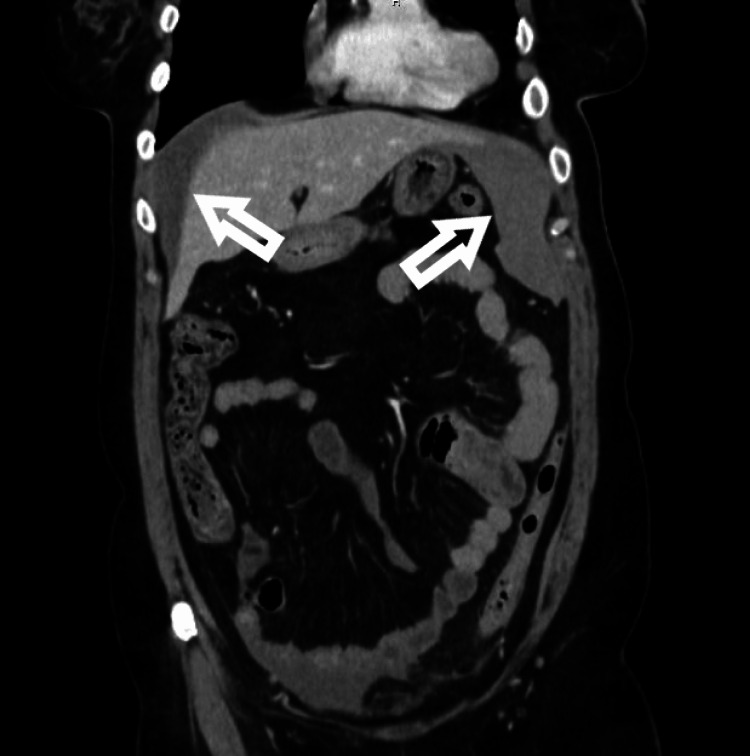
CT abdomen and pelvis with arrows pointing to free fluid in the abdomen indicating hemoperitoneum

**Figure 2:**
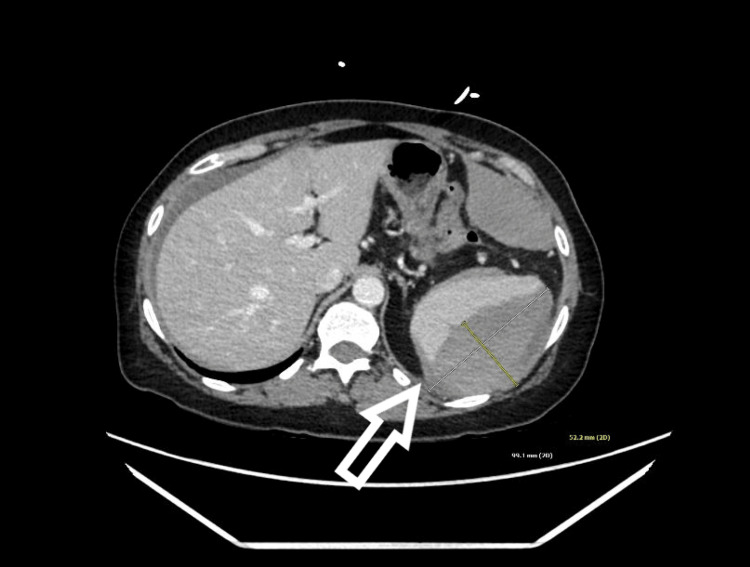
Transverse view of CT abdomen and pelvis with arrow pointing to splenic hematoma

A CT abdomen and pelvis with contrast revealed hemoperitoneum around the liver (Figure 1) and a subcapsular hematoma measuring 9.9 x 5.2cm along the lateral aspect of the spleen (Figure 2). Contrast enhancement showed there was no active extravasation but labs indicated acute blood loss with a drop in hemoglobin from 13.5 on the day of colonoscopy to 7.7 on the day of admission. While in the ED she received 2 liters of lactated ringers which resolved her hypotension and tachycardia and was admitted. 

She was treated with prophylactic immunizations for *Streptococcus pneumoniae*, *Haemophilus influenzae, *and *Neisseria meningitidis *in anticipation of likely splenectomy. General Surgery was consulted and evaluated the patient, but she became hemodynamically stable after fluids and there was no evidence of active bleeding on CT. Therefore, a decision was made to defer splenectomy at that time. Her hemoglobin was trended every 4 hours and began to slowly improve. The patient remained hemodynamically stable throughout the hospitalization. 

After improvement of the patient’s symptoms of abdominal pain and nausea, she was discharged after three days with a plan to undergo a four-week follow-up ultrasound. At that follow up, her abdominal pain was improving and her hematoma measured slightly smaller at 7.7 x 6.9 cm indicating slow resolution.

## Discussion

Colonoscopy is considered a relatively safe procedure that can be both diagnostic and therapeutic. Complications with this procedure are rare, but the most common complications include hemorrhage (.59%) as well as perforation of the colon (.07%). Splenic injury has an occurrence rate as high as one out of 5882 colonoscopies (0.017%) making it an extremely rare complication with an estimated mortality rate of 5% [8]. While the exact mechanism is unknown, potential mechanisms of splenic injury secondary to colonoscopy include excessive traction in the splenic flexure causing damage to the spleno-colic ligament, direct trauma to spleen while passing the scope through the splenic flexure and looping of the colonoscope perioperatively. Risk factors for splenic injury secondary to colonoscopy include prior abdominal surgeries causing abdominal adhesions, especially between the colon and spleen, splenomegaly, inflammatory bowel disease, pancreatitis, and polypectomy/biopsies perioperatively [15].

Following splenic injury, patients report symptoms in the first 24 hours up to 3 days later. The most common presenting symptoms in 85% of cases are generalized abdominal pain or localized left upper quadrant abdominal pain [16]. In some instances, patients present with signs of hemodynamic compromise due to acute blood loss and approximately half of patients with post-colonoscopy splenic injuries require splenectomy [16]. Though in select circumstances, such as hemodynamic stability with active bleeding found on CT, these injuries can be managed with interventional radiology performing an embolization of the bleeding vessel [17]. Our patient did not have evidence of active bleeding and was hemodynamically stable following fluid resuscitation, thus allowing for conservative medical management.

CT remains the standard for diagnosing a splenic injury in hemodynamically stable patients while ultrasound has utility in hemodynamically unstable patients. Splenic injuries are graded I-V according to the American Association for the Surgery of Trauma (AAST) scale. Using this scale, our patient had a grade III splenic injury as the hematoma’s size was >50% of the subcapsular space without evidence of active bleeding on contrasted CT. Our patient had uncomplicated Crohn’s disease and a history of multiple abdominal surgeries, which may have increased her risk for splenic injury. Her CT scan also did not show any evidence of contrast extravasation or “blush” which would have made her injury a grade IV or V and may have prompted urgent splenectomy per AAST guidelines [17].

## Conclusions

Colonoscopy remains the gold standard for diagnosing and screening for many colorectal conditions. Though no procedure is without its risk, this case highlights the importance of recognizing splenic injury as a rare but serious complication following colonoscopy, especially in patients with appropriate risk factors. Despite the low incidence of splenic injury, it is crucial for clinicians to maintain a high index of suspicion when patients present with abdominal pain after the procedure as early recognition and appropriate management could prevent the need for surgical intervention. This case could have had a much poorer outcome, but thankfully this patient’s bleeding ceased spontaneously and she was able to be managed conservatively with fluid resuscitation and monitoring. This case underscores the value of thorough follow-up and patient education on the potential risks associated with colonoscopy, ensuring prompt return to care if symptoms arise.
